# Biotechnological strategies for controlled accumulation of flavones in hairy root culture of *Scutellaria lateriflora* L.

**DOI:** 10.1038/s41598-023-47757-7

**Published:** 2023-11-21

**Authors:** Agata Wilczańska, Barbara Sparzak-Stefanowska, Adam Kokotkiewicz, Anna Jesionek, Aleksandra Królicka, Maria Łuczkiewicz, Mirosława Krauze-Baranowska

**Affiliations:** 1 Department of Pharmacognosy with Medicinal Plant Garden, Medical University of Gdańsk, Al. Gen J. Hallera 107, 80-416 Gdańsk, Poland; 2 Laboratory of Biologically Active Compounds, Intercollegiate Faculty of Biotechnology of University of Gdańsk and Medical University of Gdańsk, University of Gdańsk, A. Abrahama 58, 80-307 Gdańsk, Poland

**Keywords:** Secondary metabolism, Plant sciences, Plant biotechnology

## Abstract

Accumulation of medicinally important flavones and acteoside was evaluated in *Scutellaria lateriflora* hairy root cultures subjected to different experimental strategies – feeding with precursors of phenolics biosynthesis (phenylalanine, cinnamic acid, and sodium cinnamate), addition of elicitors (chitosan, jasmonic acid) and Amberlite XAD-4 and XAD-7 resins and permeabilization with dimethyl sulfoxide (DMSO) and methanol. The production profile of *S. lateriflora* cultures changed under the influence of the applied strategies. Hairy roots of *S. lateriflora* were found to be a rich source of wogonoside or wogonin, depending on the treatment used. The addition of sodium cinnamate (1.0 mg/L) was the most effective approach to provide high production of flavonoids, especially wogonoside (4.41% dry weight /DW/; 566.78 mg/L). Permeabilization with DMSO (2 µg/ml for 12 h) or methanol (30% for 12 h) resulted in high biosynthesis of wogonin (299.77 mg/L and 274.03 mg/L, respectively). The obtained results provide new insight into the selection of the optimal growth conditions for the production of in vitro biomass with a significant level of flavone accumulation. The data may be valuable for designing large-scale cultivation systems of hairy roots of *S. lateriflora* with high productivity of bioactive compounds – wogonin or wogonoside.

## Introduction

Many of the more than 350 species of *Scutellaria* are of medicinal importance. The biological activity of these plants has been attributed to numerous compounds acting on different targets. The most commonly reported bioactive phytochemicals in *Scutellaria* are species-specific flavones, which are present in the plant either as aglycones (such as baicalein, wogonin, scutellarein) or as 7-O-glucuronides (baicalin, wogonoside, and scutellarin, respectively) (Fig. S1)^1–5^. Flavones from *Scutellaria* plants have been shown to possess various pharmacological properties, including anti-oxidant, neuroprotective, hepatoprotective, antibacterial, antiviral, and anticancer activities, which are due to their radical scavenging ability and interaction with signalling molecules associated with autophagy, cell cycle, apoptosis, mitochondrial dynamics, inflammation, and cytoprotection^4,6–12^. In addition, the range of biological activity of acteoside (verbascoside), the phenylethanoid glycoside being an important chemotaxonomic marker of the genus, is also very wide and includes, among others, antioxidant, anti-inflammatory, immunomodulatory, analgesic, hypoglycemic, neuroprotective, and memory protective activity^13,14^.

Research on biologically active metabolites of the *Scutellaria* species seems particularly interesting in the context of the latest data showing the antiviral activity of some flavones present in this genus against the SARS-CoV-2 virus. The severity of COVID-19, the disease caused by the above mentioned coronavirus, is the result of viral infection as well as the exaggerated host immune response that amplifies pulmonary and systemic inflammations, which are the key pathological changes. The mechanism of anti-viral action of *Scutellaria* extracts and metabolites is aimed mainly at suppressing genome replication^15–18^. The studies revealed that aqueous extract of *S. barbata* D. Don, (a source of active flavonoids including wogonin), inhibited proteases involved in SARS-CoV-2 infection^15^. Flavones isolated from *S. baicalensis* (baicalein, baicalin, wogonin, norwogonin, and oroxylin A) have also been investigated as possible therapeutic substances in the treatment of SARS-CoV-2-induced acute lung injuries. With the exception of baicalin, all compounds bound to 3-chymotrypsin-like protease (3CL^pro^), which is responsible for the maturation of non-structural proteins, necessary in the life cycle of the SARS-CoV-2 virus^19^.

One of the preparations that proved effective in the treatment of COVID-19 was a Traditional Chinese Medicine preparation called Qing-Fei-Pai-Du Decoction (QFPDD), containing wogonoside among the active flavones^20,21^. It was revealed that wogonoside effectively, in a dose-dependent manner, inhibited LPS-stimulated phosphorylation of activating transcription factor-2 in RAW264.7 cells and markedly reduced the inflammatory mediators, such as IL-1β, TNF-α, and iNOS. Anti-inflammatory action of wogonoside through the ATF2 pathway may contribute to the success of QFPDD in the treatment of early-stage COVID-19 patients^20^.

One of the most studied species within the genus *Scutellaria* is *S. lateriflora* L*.* (American skullcap), which is a source of specific flavones and acteoside (Fig. S1)^1,22,23^. In nature, it is found indigenously in wetlands. The plant is native to North America and Canada where it is widespread. Besides, American skullcap is grown commercially around the world. *S. lateriflora* is used as a sedative and antispasmodic agent in the treatment of epilepsy and anxiety^1,2,24^. A particularly promising source of secondary metabolites of *S. lateriflora* are in vitro cultures^22,25–28^. Both shoots and hairy roots of this species were obtained, although they differ with respect to the levels of secondary metabolites characteristic of the genus^22,23,26,28^. Research on *S. lateriflora* has shown, that hairy roots are a more efficient source of wogonin (30 mg/g DW), wogonoside (12.0 mg/g DW), baicalin (22.54 mg/g DW) and acteoside (18.5 mg/g DW)^22,29^. Only in the case of baicalein the content determined in the shoots (6.14 mg/g DW) was higher than in hairy roots^26,28^. However, it should be emphasized that the research on shoot cultures did not take into account production, but only the content of compounds^27,28^.

Several strategies have so far been used to promote flavones’ accumulation in hairy root cultures of *S. lateriflora*. The effect of light, precursors of biosynthesis, biotic elicitors (yeast extract and bacterial lysates), methyl jasmonate, cyclodextrine, and modification of the expression of genes involved in flavonoid biosynthesis pathway, were studied among others^22,23,25–29^. The aim of this study was to investigate the rate of accumulation of medicinally important flavones and acteoside in *S. lateriflora* hairy root cultures by employing several different strategies – feeding with precursors of phenolics synthesis (phenylalanine, cinnamic acid, and sodium cinnamate), the addition of elicitors (chitosan, jasmonic acid) and in situ adsorption (Amberlite XAD-4 and XAD-7 resins). Permeabilization experiments were also performed with the use of DMSO and methanol. Additionally, the preliminary study was carried out to assess the viability of scaling-up biomass production using the basket-bubble bioreactor and to examine the possibility of using *Pectobacterium carotovorum* lysate for large-scale production of flavones.

## Materials and methods

All the methods were performed in accordance with relevant guidelines and regulations.

### Chemical reagents

Acteoside, baicalin, baicalein, wogonin, and chrysin were purchased from Extrasynthese (Genay, France). Scutellarin and wogonin 7-O-glucuronide (wogonoside) were from Phytomarker (Tianjin, China). All other chemicals were standard commercial products of analytical grade. All reagents used for cultivation of *S. lateriflora* hairy roots were declared to have proper quality for in vitro cultures (Sigma-Aldrich, St. Louis, MO, United States).

### Plant material and initiation of shake flask cultures of hairy roots of *S*. *lateriflora*

In the study, the previously established hairy roots of *S. lateriflora* served as a source of plant material. Their origin, initiation, and confirmation of transformation were fully described previously^22^. Briefly, the hairy roots were induced from *S. lateriflora *in vitro grown seedlings and after transformation with *Agrobacterium rhizogenes* A4 (ATCC 31798), they were maintained on hormone-free Gamborg medium^30^, containing 3% (*w*/*v*) sucrose, with half-strength micro- and macro-elements (further referred as 1/2 B5 medium), in the dark in 100 ml Erlenmeyer flasks containing 50 ml of the above medium (Fig. S2A). The hairy roots were subcultured every 40 days.

### Evaluation of biomass growth

Hairy root cultures of *S. lateriflora*, grown in the course of the study, were evaluated for fresh weight (FW) content (expressed in g/L). Subsequently, the harvested biomasses were freeze-dried in order to assess their dry weight (DW), expressed in g/L. Growth factor (Gf) of the examined cultures was calculated according to the formula: Gf = [(FW_final_–FW_initial_)/FW_initial_] × 100%, where FW_final_ is the fresh weight of biomass at the end of the experiment and FW_initial_ is the weight of the inoculum. The “growth rate” was expressed as fold increase of FW in the course of the experiment.

### Precursors of phenolics synthesis preparation and treatment

Phenylalanine, cinnamic acid and sodium cinnamate (Sigma-Aldrich, St. Louis, MO, United States) were dissolved in redistilled water (100 mg in 100 ml). The solutions of biosynthetic precursors were added by membrane filtering (a 25 mm diameter sterile syringe filter with a 0.2 µm pore size hydrophobic PTFE membrane) to the hairy root culture in the initial phase (0 day) or in the stationary phase (26 day) of the growth cycle (final concentration of phenylalanine in the culture: 0 day – 0.1, 0.2, 0.4, 0.6, 0.8 and 1.0 mmol/L, 26 day – 0.1 and 0.4 mmol/L; final concentration of cinnamic acid: 0 day – 1, 5 and 10 mg/L; sodium cinnamate: 0 day and 26 day – 1, 5, 10 and 25 mg/L). The corresponding volume of sterilized redistilled water was added to the control groups. The biomass was collected on the 40th day of the growth cycle.

### Permeabilization solutions preparation and treatment

As permeabilizing agents, dimethyl sulfoxide (DMSO) in concentrations of 1, 2 and 10 µg/ml and methanol in concentrations of 10, 20 and 30% (of the culture volume) were added by membrane filtering to the culture medium, on the 26th day of the growth cycle for 12 and 24 h. After this time, the growth medium was collected and submitted for chromatographic analysis and the culture was replenished with 50 ml of fresh, 1/2 B5 medium. The control groups were: 1.Hairy roots cultivated for 40 days, without the addition of the permeabilizing agents 2. Hairy roots grown without the addition of the permeabilizing agents, for 40 days, with the medium replenished on the 26th day. All biomasses were collected on the 40th day of the growth cycle.

### Amberlite XAD-4 and XAD-7 resins preparation and treatment

The Amberlite XAD-4 and XAD-7 resins (Rohm and Haas, France) were washed with ethanol (3 × 100 ml) followed by redistilled water (3 × 100 ml), then placed in sachets made of sterile gauze, later referred to as "mini-bags" (50, 100, 500 and 1000 mg per bag), and steam sterilized (121 °C, 0.1 MPa, 20 min). Approx. 1.0 g of biomass was inoculated in 50 ml of a liquid 1/2B5 medium, in 100 ml Erlenmeyer flasks, equipped with the "mini-bags" in the initial phase (day 0). The hairy roots cultured in the medium without the "mini-bags" were the control group. All biomasses were collected on the 40th day of the growth cycle.

### Elicitors preparation and treatment

Chitosan (Sigma-Aldrich, St. Louis, MO, United States) was dissolved in 1% acetic acid (100 mg in 100 ml), neutralized with 0.1 N NaOH and steam sterilized (121 °C, 0.1 MPa, 20 min). It was added to the medium on the 26th day of the growth cycle at concentrations of 30, 100, 200 and 250 mg/l for 7 and 14 days, and at concentrations of 30 mg/l for 24, 48, 72 and 96 h. Two control groups were included: the non-treated hairy roots cultivated under standard conditions, as well as cultures supplemented with corresponding volume of acetic acid neutralized with 0.1N NaOH.

Jasmonic acid (Sigma-Aldrich, St. Louis, MO, United States) was dissolved in ethanol (1 mg/ml) and added to the medium by membrane filtering at concentrations of 100, 200, and 300 µmol/l, on the 26th day of the growth cycle. The exposure time was 7 and 14 days. Two control groups were included – the non-treated hairy roots cultivated under standard conditions or cultures grown with the addition of the corresponding volume of ethanol. Both in the case of elicited and control cultures, the biomasses were collected at the end of the elicitation period.

### Hairy roots of *S*. *lateriflora* cultured in a bioreactor

Hairy roots of *S. lateriflora* (ca 10 g) were placed evenly in the basket of a basket-bubble bioreactor, described in detail in the previous work^31^, and fully immersed in 1/2 B5 medium (1000 ml). The aeration rate was about 670 ml/min. The ratio of *inoculum* to the volume of growth medium was 1:100 (*m*/*v*). The biomass was cultivated in the dark, for 20, 40 and 60 days, to determine the culture growth parameters: fresh weight FW (g/L), dry weight DW (g/L), and growth factor Gf (%), and the metabolic profile.

The origin and preparation of bacterial suspension of *Pectobacterium carotovorum* used for elicitation of the bioreactor-grown hairy roots of *S. lateriflora* has been described in earlier work^22^. Bacterial suspension of *P. carotovorum* was added to the medium at the concentration of 15 ml/L on the 40th day of the experiment. The biomass was collected on the 60th day of the growth cycle.

### Analysis of secondary metabolites (HPLC)

The extraction and analysis of the investigated compounds was performed according to the previously developed HPLC method^22^ (Fig. S3). Briefly, HPLC was performed on a C18 (2.6 µm) column (100 × 2.1 mm) using gradient elution with increasing concentration of mobile phase B (acetonitrile/water/trifluoracetic acid; 1:1:0.01, *v*/*v*) from 20 to 100% in mobile phase A (water/trifluoracetic acid, 1:0.01 *v*/*v*) (0 min – 20% B, 30 min – 40% B, 60 min – 80% B, 70 min – 100% B) all at 0.2 ml/min. Detection was carried out at 280 nm.

The HPLC method was validated in terms of linearity, repeatability, intra- and inter-day precision, limit of quantification (LOQ), and recovery (Tables S1, S2).

The determined amounts of analytes were presented as “content “(expressed in mg/g DW), “production” (expressed in mg/L) and, “productivity” (expressed in mg/L/day).

### Statistical analysis

All data are the mean of three independent experiments (n = 3). Statistical analysis was carried out using Student’s *t* test or Mann–Whitney test. Probability of *p* < 0.05 was considered significant. Analysis was performed using the SigmaStat 3.5 program (Statcon, Germany). The results of bioreactor experiments are considered as preliminary (n = 1).

## Results

### Precursor feeding

#### Phenylalanine

**Figure 1:**
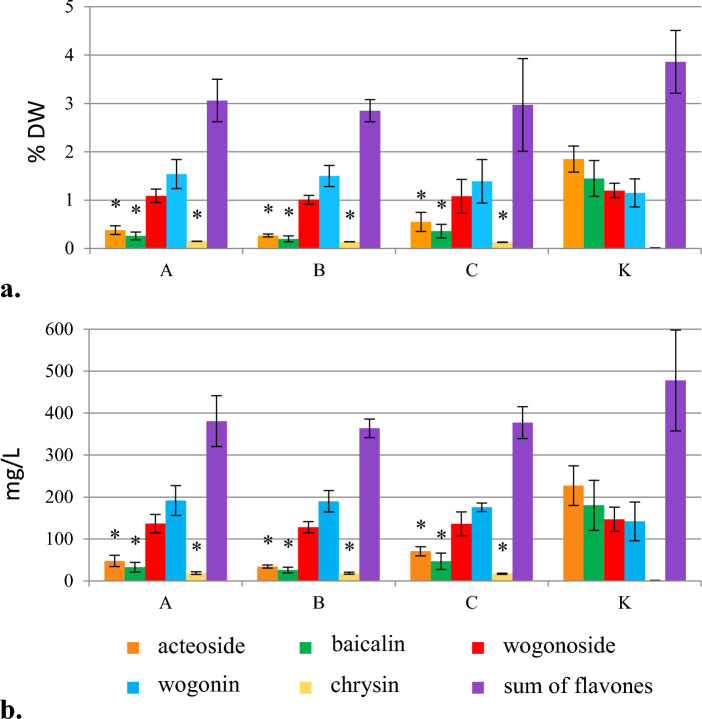
The effect of phenylalanine added in the initial or the stationary phase of *S. lateriflora* hairy root culture on the content (% DW) (**a**) and production (mg/L) (**b**) of acteoside and flavones in the biomass, cultivated for 40 days (mean values, n = 3 ± SD). The values marked with * are statistically significant compared with the control at p < 0.05. A—In the initial phase of culture at a concentration of 0.1 mmol/L, B—In the stationary phase of the culture at a concentration of 0.1 mmol/L, C—In the stationary phase of the culture at a concentration of 0.4 mmol/L, K—hairy roots grown under standard conditions (control).

Phenylalanine added on the day of inoculation at concentrations above 0.1 mmol/L inhibited the growth of *S. lateriflora* hairy roots. However, the addition of this precursor in the stationary phase of the culture did not affect the growth parameters regardless of the concentration used (Fig. S4). A decrease in the production of acteoside and flavone glycoside – baicalin, and an increase in the accumulation of aglycone – chrysin was observed, which was about 15 times higher than in the control group (Fig. 1). It should be noted, however, that compared with other flavones, the content of chrysin was still low (0.15% DW) (Fig. 1, Table 1). The studied biomass did not produce scutellarin or baicalein. The content of wogonin and wogonoside was unchanged, as compared with the control sample.

**Table 1:** The content and production of acteoside and flavones in *S. lateriflora* hairy root cultures grown in shake flasks and subjected to various biotechnological strategies.

Compound	Experimental strategy																	
	Phenylalanine^a^		Cinnamic acid^b^		Sodium cinnamate^c^		DMSO^d^		Methanol^e^		XAD-7^f^		Chitosan^g^		Jasmonic acid^h^		Reference culture^i^	
	% DW	mg/L	% DW	mg/L	% DW	mg/L	% DW	mg/L	% DW	mg/L	% DW	mg/L	% DW	mg/L	% DW	mg/L	% DW	mg/L
Acteoside	0.38	47.83	0.73	71.48	1.97	253.09	1.06	142.98	0.06	4.99	1.42	133.34	2.1	223.86	2.39	208.52	1.85	227.2
Scutellarin	–	–	–	–	–	–	–	–	–	–	0.20	18.78	–	–	–	–	0.06	7.1
Baicalin	0.26	32.84	1.39	136.19	2.02	259.00	1.03	137.53	0.05	4.39	1.28	120.19	0.68	72.49	2.12	245.77	1.45	180.3
Wogonoside	1.09	136.59	2.75	270.58	4.41	566.78	0.83	112.29	0.04	3.78	0.95	89.21	3.16	336.86	2.57	310.60	1.20	147.1
Baicalein	–	–	–	–	–	–	–	–	–	–	–	–	–	–	–	–	–	–
Wogonin	1.54	191.68	0.69	67.67	0.32	41.05	2.11	282.45	3.11	274.03	0.99	92.96	0.20	21.32	0.33	41.20	1.15	142.0
Chrysin	0.15	18.6	–	–	–	–	0.17	21.2	0.24	20.69	–	–	–	–	–	–	0.01	1.3
Sum of flavones^*j*^	3.06	380.96	4.87	481.95	6.75	868.55	4.13	553.47	3.44	302.89	3.44	323.02	4.04	469.04	5.05	598.87	3.86	477.8

^a^0.1 mmol/l, added on day 0 of the growth cycle (biomass grown for 40 days).

^b^5 mg/l, added on day 0 of the growth cycle (biomass grown for 40 days).

^c^1 mg/l, added on 26th day of the growth cycle (biomass grown for 40 days).

^d^Dimethyl sulfoxide, 10 µg/ml for 24 h, added on 26th day of the growth cycle (biomass grown for 40 days).

^e^30% for 12 h, added on 26th day of the growth cycle (biomass grown for 40 days).

^f^50 mg/50 ml, added on day 0 of the growth cycle (biomass grown for 40 days).

^g^30 mg/l for 14 days, added on 26th day of the growth cycle (biomass grown for 40 days).

^h^300 µmol/l for 7 days, added on 26th day of the growth cycle (biomass grown for 33 days).

^i^Biomass grown for 40 days, data according to^22^.

^j^The sum of six flavones: baicalin, wogonoside, wogonin, baicalein, scutellarin and chrysin.

#### Cinnamic acid

**Figure 2:**
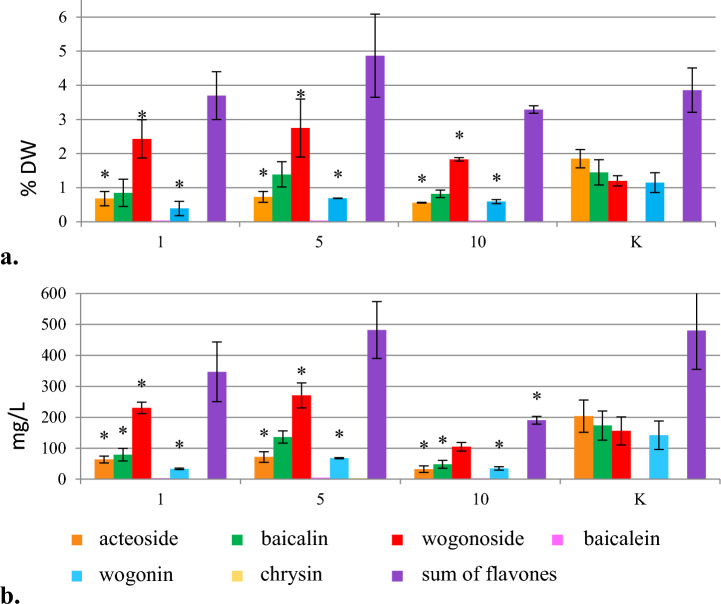
The effect of cinnamic acid added at concentrations of 1.0, 5.0 and 10.0 mg/L in the initial phase of *S. lateriflora* hairy root culture on the content (% DW) (**a**) and production (mg/L) (**b**) of acteoside and flavones in the biomass, cultivated for 40 days (mean values, n = 3 ± SD). The values marked with * are statistically significant compared with the control at p < 0.05. K—hairy roots grown under standard conditions (control).

The addition of cinnamic acid at a concentration of 10 mg/L inhibited the growth of hairy roots, which was about 2 times lower than in the control (Fig. S5). As a result of cinnamic acid supplementation, the accumulation of wogonoside increased, in contrast to the production of acteoside and wogonin, which was more than twofold lower than in the control (Fig. 2). Baicalin content remained at the control level. The highest content and production of wogonoside (2.75% DW and 270.58 mg/L, Table 1), which were 2.3- and 1.9-fold higher than in the control, were found after adding 5.0 mg/L cinnamic acid.

#### Sodium cinnamate

**Figure 3:**
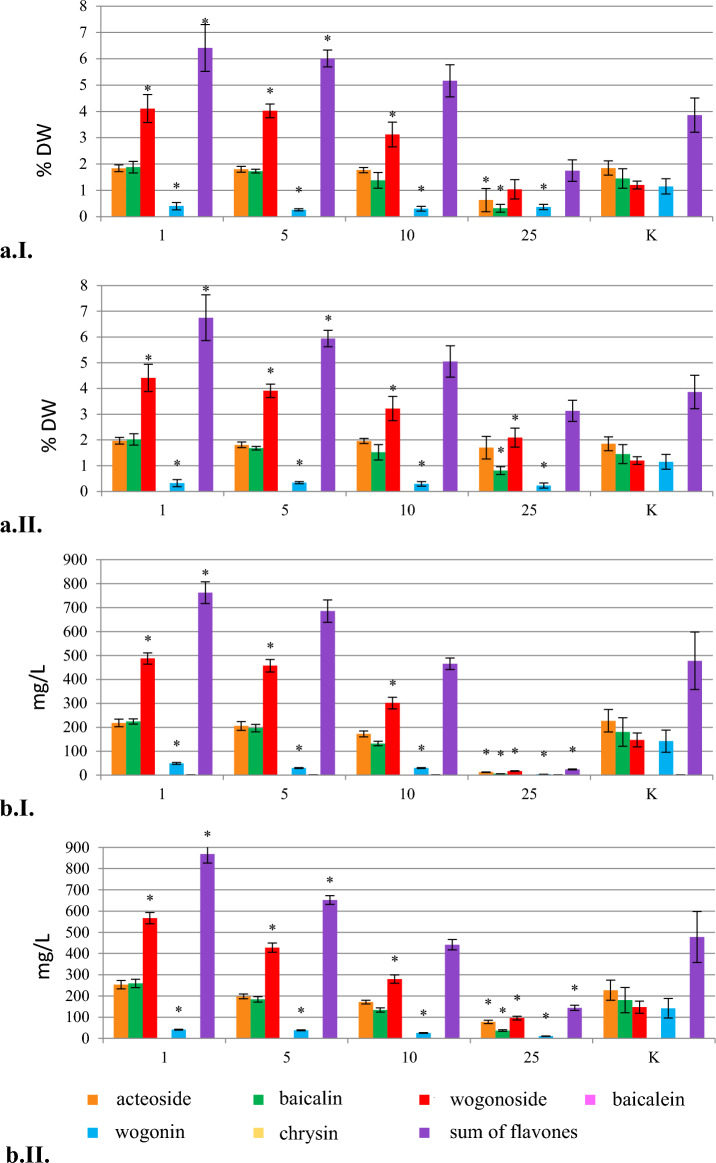
The effect of sodium cinnamate added at concentrations of 1.0, 5.0, 10.0 and 25.0 mg/L mg/L in the initial phase (**I**) and in the stationary phase (**II**) of *S. lateriflora* hairy root culture on the content (% DW) (**a**) and production (mg/L) (**b**) of acteoside and flavones in the biomass, cultivated for 40 days (mean values, n = 3 ± SD). The values marked with * are statistically significant compared with the control at p < 0.05. K—hairy roots grown under standard conditions (control).

The growth of hairy roots after sodium cinnamate treatment was similar to that of the control, but as the precursor concentration increased, it was inhibited, until complete necrosis, which occurred at 25.0 mg/L (Fig. S6). It was found out that the addition of 1.0 mg/L sodium cinnamate, during the stationary phase of culture, was the most effective and resulted in the highest total content and production of flavonoid compounds (6.75% DW and 868.55 mg/L) (Table 1), which were 1.8 times higher compared with the control. Under the same conditions, the highest content (4.41%) and production (566.78 mg/L) of a single metabolite – wogonoside (Table 1), which were almost four times higher than in the control group (Fig. 3), were achieved. Sodium cinnamate did not inhibit acteoside biosynthesis. The addition of this precursor either on the inoculation day or during the stationary phase, caused a threefold decrease in wogonin production. The hairy root cultures supplemented with sodium cinnamate did not produce scutellarin, whereas baicalein content corresponded to that of the control sample.

### Permeabilization with DMSO and methanol

**Figure 4:**
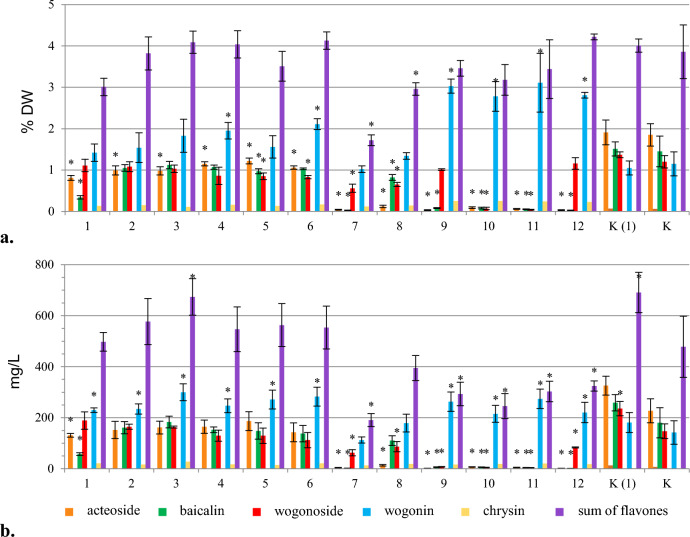
The effect of permeabilizing factors, DMSO and methanol, on the content (% DW) (**a**) and production (mg/L) (**b**) of acteoside and flavones in the *S. lateriflora* hairy root culture (mean values, n = 3 ± SD). The values marked with * are statistically significant compared with the control at p < 0.05. The values denoted by ^ are statistically significant compared with the control with the exchanged medium at p < 0.05. (1) and (2) DMSO at a concentration of 1 μg/ml for 12 and 24 h, respectively; (3) and (4) DMSO at a concentration of 2 μg/ml for 12 and 24 h, respectively; (5) and (6) DMSO at a concentration of 10 μg/ml for 12 and 24 h, respectively; (7) and (8) methanol at a concentration of 10% for 12 and 24 h, respectively; (9) and (10) methanol at a concentration of 20% for 12 and 24 h, respectively; (11) and (12) methanol at a concentration of 30% for 12 and 24 h, respectively; K (1)—hairy roots grown in a system with replacement of the culture medium; K—hairy roots grown under standard conditions (control).

The analysis of the control samples showed that medium replenishment significantly improved growth parameters of the hairy root culture. The increase in the biomass, achieved with the addition of the fresh medium, was over 24-fold compared with about 13-fold increase recorded for the biomass grown under standard conditions. Replenishment of the culture medium stimulated not only the growth but also the production of secondary metabolites in the culture (Fig. S7). The content of acteoside and flavones in the control cultures, maintained with or without medium replenishment, remained at a similar level. However, due to the stimulating effect of medium exchange on biomass growth, the total production of flavones in the culture was 1.4 times higher – 690.5 mg/L compared with 477.80 mg/L in the culture grown under standard conditions (Fig. 4).

The addition of DMSO had no effect on culture growth, which was comparable to the control with medium replenishment, except for the use of the highest concentration of DMSO for a period of 24 h. In contrast to DMSO, the addition of methanol (for tested concentrations and permeabilization time of 12 and 24 h) resulted in a deterioration of biomass growth, which became dark brown and brittle (Fig. S7). Under the influence of both factors, change in the colour of culture media was observed, and it was the most intense in the case of using the highest concentration of methanol (30%). In contrast to the biomass grown in the presence of DMSO, the root cultures treated with methanol were characterized by a different production profile. At the highest methanol concentrations (20 and 30%), the root cultures selectively accumulated wogonin and, in small amounts, wogonoside and chrysin (Fig. 4).

In all permeabilized biomasses, no increase in the total content of flavonoids was observed in comparison to both control groups, i.e. the roots cultivated in standard conditions and in the system with the replenished medium (Fig. 4). Under the influence of DMSO used at concentrations of 2 and 10 µl/ml for 24 h and methanol at concentrations of 20 and 30%, both for 12 and 24 h, an increase in the content of wogonin in the examined root cultures was observed.

Using DMSO at a concentration of 10 µl/ml for 24 h, the highest concentration of wogonin was achieved (2.11% DW, 1.83-fold increase) (Table 1), while the use of DMSO at a concentration of 2 µl/ml for 12 h resulted in the highest production of the above-mentioned compound (299.77 mg/L, 2.11-fold increase). Interestingly, under the influence of methanol added at higher concentrations (20–30%), the biomass changed the production profile and selectively accumulated wogonin, together with very low amounts of wogonoside and chrysin. The highest content and production of wogonin (3.11% and 274.03 mg/L) (Table 1), which was 2.7 and 1.9 times higher than in the control, respectively, was confirmed in the biomass treated with methanol at the concentration of 30% for 12 h. Under the influence of DMSO and methanol, the concentration of acteoside in hairy roots was also reduced. No scutellarin or baicalein were found in the permeabilized roots. As a result of permeabilization, the biomass released into the medium only two flavonoids, wogonin and chrysin. The presence of wogonin in the culture medium was revealed after permeabilizaton with methanol (10–30%) and DMSO (1 µl/mL), while chrysin was detected only after the addition of methanol. The medium was characterized by the highest content of both chrysin and wogonin after adding methanol at a concentration of 30% for 12 h (0.58 mg/L of chrysin and 69.06 mg/L of wogonin). The total production of wogonin in the developed system (30% MeOH, 12 h) was 343.09 mg/L (274.03 mg/L + 69.06 mg/L) and was 2.4 times higher than in the parent culture.

### Addition of Amberlite XAD-4 and XAD-7 resins

**Figure 5:**
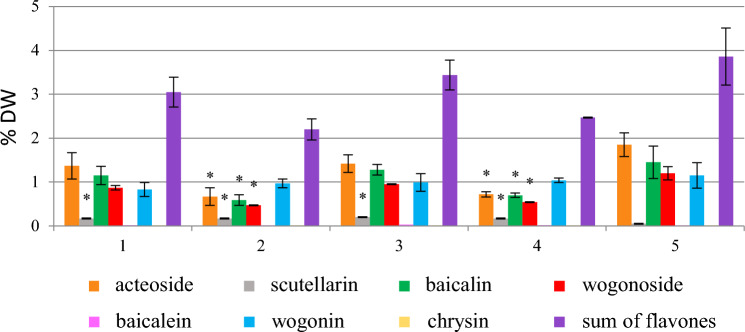
The effect of Amberlite XAD-4 and XAD-7 resins used in the form of "mini-bags" at a concentration of 50 mg/50 ml on the content (% DW) of acteoside and flavones in *S. lateriflora* hairy root culture (mean values, n = 3 ± SD). The values marked with * are statistically significant compared with the control at p < 0.05. 1 – biomass cultivated in the presence of XAD-4 resin in the form of "mini-bags"; 2 – "mini-bags" with XAD-4 resin; 3 – biomass cultivated in the presence of XAD-7 resin in the form of "mini-bags"; 4 – "mini-bags" with XAD-7 resin; K—hairy roots grown under standard conditions (control).

The growth parameters of *S. lateriflora* hairy roots were not affected by the presence of Amberlite XAD-4 and XAD-7 resins, applied in the form of the "mini-bags" (Fig. S8). The production profile of hairy roots also did not change, and the dominant compounds in the biomass were acteoside and glycoside forms of flavones – wogonoside and baicalin. A decrease in the accumulation of all analyzed compounds was observed, except for scutellarin, whose content and production were about 3 times higher than in the control sample (Fig. 5).

HPLC analysis of the extracts obtained from the "mini-bags" showed that flavones and acteoside were adsorbed by the resins. Among the analyzed compounds, wogonin was selectively released into the medium. Compared with the content of the hairy roots, wogonin was the dominant compound in the “minibags” and its content was 1%. As a result of the lower content of acteoside and flavones in hairy roots grown in the presence of the "mini-bags" with XAD-4 and XAD-7 resins, the total content of the investigated compounds in the biomass and the "mini-bags" was comparable to the control sample.

### Elicitation

#### Chitosan

The effect of chitosan was tested after adding it to a hairy root culture of *S. lateriflora* in the stationary phase (day 26 of the growth cycle) for a short period of time (24 to 96 h)^32,33^, or subjected to its effects for an extended period (from 7 to 14 days)^34^. In a short-term experiment, in which 30 mg/L of chitosan was added for 24, 48, 72 and 96 h, no effect on biomass growth was observed (Fig. S9). Also, no significant differences were found in the parameters of biomass growth and the levels of analysed secondary metabolites in the hairy root culture treated with neutralized acetic acid (one of the control groups). In the extended elicitation experiment, after 7 and 14 days of elicitation, low concentrations of chitosan (30 and 100 mg/L) had no effect on the growth of the root culture. However, the use of higher concentrations of the elicitors (200 and 250 mg/L) caused hairy root necrosis.

**Figure 6:**
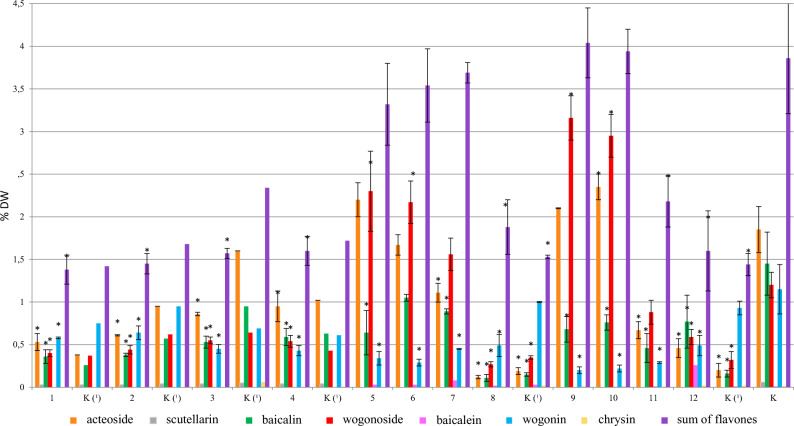
The effect of chitosan added in the stationary phase of *S. lateriflora* hairy root culture on the content (% DW) of acteoside and flavones in the biomass (mean values, n = 3 ± SD). The values marked with * are statistically significant compared with the control cultivated under standard conditions at p < 0.05. Chitosan added: at a concentration of 30 mg/L – biomass collected after 24 h (1), 48 h (2), 72 h (3), 96 h (4), 7 days (5) and 14 days (9); at a concentration of 100 mg/L – biomass collected after 7 days (6) and 14 days (10); at a concentration of 200 mg/L – biomass collected after 7 days (7) and 14 days (11); at a concentration of 250 mg/L – biomass collected after 7 days (8) and 14 days (12); K (1) – hairy roots grown after addition of a specified volume of acetic acid neutralized with 1 N NaOH at the appropriate time of the experiment; K – hairy roots grown under standard conditions (control).

Both after 7 and 14 days from the addition of chitosan, an inverse relationship was found between the increase in elicitor concentration and the ability of biomass to biosynthesise acteoside and flavones. For chitosan added at concentrations 30, 100 and 200 mg/L, both 7 and 14 days after elicitation, the dominance of wogonoside was observed. Elicitation at the concentration of 30 mg/L for 14 days most significantly increased the accumulation (2.7-fold, 3.2%) and production (2.3-fold, 336.86 mg/L) of wogonoside (Fig. 6) (Table 1). On the other hand, the content of baicalin and wogonin in the elicited biomass was approximately 2- and threefold lower, respectively, compared with the control, with an unchanged level of acteoside accumulation. The roots elicited with chitosan at the highest concentration (250 mg/L) were distinguished by about 3 times lower content of all analyzed compounds compared with the standard control. In contrast to chitosan, the control samples treated with neutralized acetic acid were characterized by increased accumulation of wogonin compared with the remaining compounds. However, its concentrations (about 1%) were at the level of the parent culture.

#### Jasmonic acid

**Figure 7:**
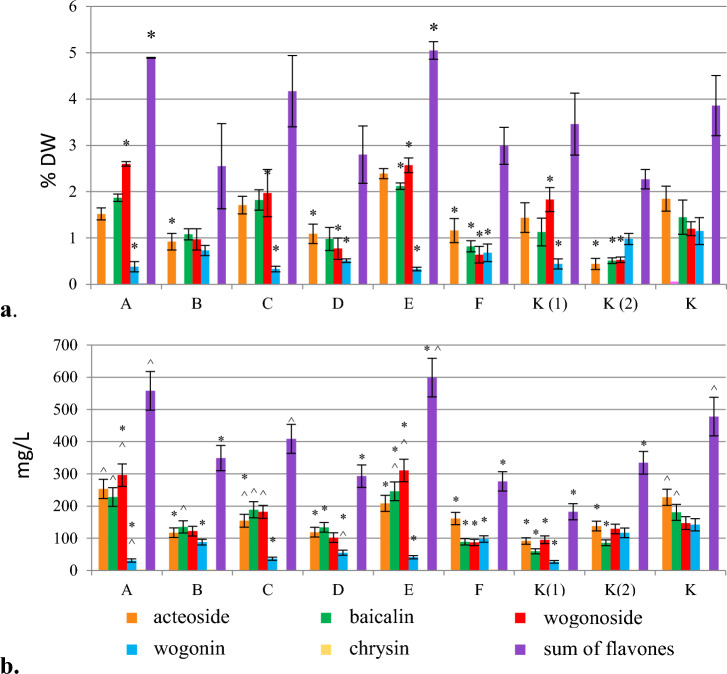
The effect of jasmonic acid added in the stationary phase of *S. lateriflora* hairy root culture on the content (% DW) (**a**) and production (mg/L) (**b**) of acteoside and flavones in the biomass (mean values, n = 3 ± SD). The values marked with * are statistically significant compared with the control cultivated under standard conditions at p < 0.05. The values marked with ^ are statistically significant compared with the control cultivated in the presence of ethanol at p < 0.05. Jasmonic acid added: at a concentration of 100 µmol/L – biomass collected after 7 days (A) and 14 days (B); at a concentration of 200 µmol/L – biomass after 7 days (C) and 14 days (D); at a concentration of 300 µmol/L – biomass collected after 7 days (E) and 14 days (F); K (1) – hairy roots grown in the presence of ethanol and collected after 7 days; K (2) – hairy roots grown in the presence of ethanol and collected after 14 days; K – hairy roots grown under standard conditions (control).

The growth parameters of hairy roots, collected 7 days after the addition of jasmonic acid, suggested an inhibitory effect of ethanol on the growth of the biomass, however, the same effect has not been confirmed within a 14-day time period (Fig. S10). Baicalin and wogonoside, i.e. the glycoside forms of flavones, were the dominant compounds. Their highest contents (2.12%, 1.5-fold increase vs. control, and 2.57%, 2.1-fold, respectively) and production rates (245.77 mg/L, 1.3-fold, and 310.6 mg/L, twofold, respectively) were determined after the addition of 300 µmol/L jasmonic acid to the culture, which was then collected 7 days after elicitation (Fig. 7, Table 1). The amount of acteoside remained unchanged, but a reduced content of wogonin was observed. In all biomasses elicited with jasmonic acid or cultured in the presence of ethanol, a decrease in the accumulation of the analyzed compounds was recorded between 7 and 14 days after elicitation.

### Bioreactor experiments

#### Bioreactor-grown hairy roots

In the 60-day experiment, the hairy roots of *S. lateriflora*, cultivated in the basket-bubble bioreactor on 1/2B5 medium (Fig. S2B,C), showed continuous and intensive growth (ca. 20-fold increase in biomass concentration) (Fig. S11). Bioreactor-grown hairy roots were found to be a rich source of wogonoside, whose content (3.49%) and production (659.44 mg/L) were 2.9 times and 4.5 times higher, respectively, than in the biomass grown as shake culture. The production of flavones, determined after 60 days, was 934.42 mg/L and it was almost twice as high as in the shake culture (Table S3).

#### Elicitation with *Pectobacterium carotovorum* lysate

Elicitation with *P. carotovorum* bacterial lysate slightly limited the growth of the culture (16-fold increase *vs*. 20-fold increase in bioreactor-grown hairy roots under standard conditions), probably by reducing the hydration of root tissues, since the dry mass determined for both cultures was similar (about 19 g/L). G_f_ was 1867.61%, while fresh and dry weight was – 300.76 g/L and 18.91 g/L, respectively.

HPLC analysis showed that after the elicitation with *P. carotovorum*, the production profile of the bioreactor-grown culture changed (Table S3). The dominant compound was wogonin, the content (3.79%) and production (761.17 mg/L) of which were 3.3- and 5.4-fold higher, respectively, than in the shake culture. The elicited biomass also accumulated larger amounts of scutellarin (48.59 mg/L) and free aglycones: baicalein (42.51 mg/L) and chrysin (8.1 mg/L). The total production of flavones (1074.94 mg/L) in the elicited roots was 1.4-fold higher than in the parent culture grown in the bioreactor and 2.7-fold higher than in the shake culture (Table S3).

In order to compare the hairy roots grown in the shake flasks and in the bioreactor in terms of the efficiency of secondary metabolites accumulation, the productivities of the analyzed compounds (expressed in mg/L/day) were calculated (Table S3). In *S. lateriflora* biomass grown in the bioreactor under standard conditions, the highest productivity of wogonoside (10.99 mg/L/day) was achieved. In the bioreactor-grown hairy roots treated with *P. carotovorum*, the highest productivity of baicalein (0.71 mg/L/day), chrysin (0.14 mg/L/day), and wogonin (12.67 mg/L/day) as well as the highest total flavone yield (17.91 mg/L/day) was observed (Table S3).

## Discussion

Various strategies aimed at increasing the accumulation of biologically active secondary metabolites have been used for in vitro cultures of several *Scutellaria* species^22,26,35–37^. The presented work includes research on a number of retro-biosynthetic approaches that have not been applied so far in the hairy root culture of *S. lateriflora*. Transgenic hairy roots have been investigated intensively for their ability to induce stable, high-rate production of secondary metabolites, which is genetically controlled, but also influenced by nutritional and environmental factors^22,29^. In the conducted study, the rate of biosynthesis of medicinally important flavones and acteoside was evaluated in *S. lateriflora* hairy roots subjected to different biotechnological strategies, including supplementation of precursors of phenolic biosynthesis (phenylalanine, cinnamic acid and sodium cinnamate), the addition of elicitors (chitosan, jasmonic acid), and Amberlite XAD-4 and XAD-7 resins, as well as permeabilization with DMSO and methanol. Moreover, the preliminary results of the conducted scale-up study were also taken into account. The initial concentrations of the analyzed metabolites and the productivity of the parent culture of *S. lateriflora* grown on 1/2B5 medium were established previously^22^. The following values were determined respectively for the content and productivity of individual compounds: acteoside – 18.5 mg/g, 227.2 mg/L; scutellarin – 0.6 mg/g, 7.1 mg/L, baicalin – 14.5 mg/g, 180.3 mg/L; wogonoside – 12.0 mg/g, 147.1 mg/L; wogonin – 11.5 mg/g, 142.0 mg/L, chrysin – 0.1 mg/g, 1.3 mg/L and 38.6 mg/g and 477.8 mg/L for sum of flavones^22^. It was observed that as a result of the experiments, the production profile changed depending on the cultivation system applied. However, the dominant compounds, next to baicalin and acteoside, were always wogonoside or wogonin alternately.

The addition of biosynthesis precursors of plant phenolics to culture media is a commonly applied strategy, used to increase the level of production of secondary metabolites in in vitro plant cultures^38,39^. Phenylalanine – the amino acid and phenylpropanoids precursor, is an intermediate compound in the biosynthesis of cinnamic acid, which is then transformed into flavones in plant cells^40^. In the studies performed, feeding with cinnamic acid and sodium cinnamate significantly increased the accumulation of wogonoside (wogonin 7-O-glucuronide), while after the addition of phenylalanine, depending on individual biologically active metabolites, their content was lower or remained unchanged compared with the control sample (Figs. 1, 2 and 3). In contrast to sodium cinnamate, the hairy root culture of *S. lateriflora*, both in the presence of phenylalanine and cinnamic acid, was characterized by a significantly reduced ability to biosynthesize acteoside (Figs. 2, 3).

The results of the presented experiments on *S. lateriflora*, involving the use of precursors of biosynthesis, are consistent with the results obtained for root cultures of other *Scutellaria* species. In the study on the accumulation of flavonoids in the transformed roots of *S. baicalensis* performed by Kuzovkina^37^ the addition of 0.01–1.0 mM of phenylalanine to the nutrient medium affected neither the growth of the roots nor the content flavonoids in them. This precursor also turned out to be ineffective in the study aimed at evaluating its effect on the levels of baicalin and baicalein in suspension and callus cultures of *S. baicalensis*^41^, while sodium cinnamate (5 mg/L) and cinnaminic acid (1 mg/L) were the most effective.

Studies on shoot cultures showed that the effects of precursor supplementation depended on the cultivation method used. In agar cultures of *S. lateriflora* shoots, supplemented with phenylalanine (1 g/L), the total flavonoid content was lower or equal to the control sample with the strongest decrease of wogonoside concentration. On the other hand, in agitated shake flask cultures, the addition of phenylalanine at a concentration of 1–2.5 g/L improved total flavonoid concentration, with the highest content of 3764.8 mg/100 g (2.24-fold increase compared with the control)^28^.

Permeabilization is the process of increasing the permeability of cytoplasmic membranes for secondary metabolites, through disintegration or dissolution of the lipid fraction of the membrane by various chemical and physical factors. This process is of practical importance because it enables the release of intracellulary stored metabolites into the medium, which makes them easier to recover by liquid–liquid extraction of culture media or the use of resins^42–44^. So far, the effect of permeabilization on in vitro cultures of any of *Scutellaria* species has not been studied. The most commonly used permeabilizing agent is dimethyl sulfoxide (DMSO)^42–46^, whereas methanol, on the other hand, is much less frequently used^47^. It has been revealed that permeabilization with these agents, applied to *S. lateriflora* hairy roots for the first time in the current work, can be used to increase the concentration of wogonin in the biomass, as well as in the culture medium. Unlike methanol, the addition of DMSO generally did not affect culture growth. It is worth noting that the exchange of the culture medium in the stationary phase improved biomass growth parameters (Fig. S7). It should be emphasized that the total production of wogonin in the developed system (30% MeOH, 12 h) was 343.09 mg/l (274.03 mg/L + 69.06 mg/L – the sum calculated for biomass production and the content in the collected medium), which is 2.4 times higher than in the parent culture. Therefore, the described system can be considered as a rich source of wogonin. Noteworthy is also the observed increase of chrysin production in the roots (Fig. 4). Contrary to the control culture, the above compound was also present in the growth medium. The developed system enables to optimize the downstream processes by obtaining wogonin in a relatively simple way, due to selective release of the above compound into the culture medium.

The solid adsorbents such as Amberlite resins (XAD-4 and XAD-7) can stimulate de novo synthesis of secondary metabolites, removing some of them from the biomass. Moreover, they can also protect natural compounds against degradation^43^. These are polymers characterized by a highly porous structure, whose inner surface can adsorb non-covalently and non-ionically, and then release various chemical compounds in the elution process. Amberlite XAD resins are also known for eliciting properties^44,48,49^.

Preliminary studies showed that adding polymer resins directly to the culture medium had a negative effect on the growth and viability of *Scutellaria* root cultures (unpublished data). Therefore, in our studies, the addition of XAD-4 and XAD-7 resins in the form of so called "mini-bags" was used. In the conducted experiment, no differences were found in the effect of both types of resins on the biosynthesis of secondary metabolites. Also, the total content of the tested compounds in the biomass and in the "mini-bags" was comparable to the control sample. HPLC analysis of the extracts obtained from the "mini-bags" showed that flavones and acteoside were adsorbed by the resins, thus behaving similarly as in the previous study on root culture of *S. barbata*^49^. It seems, that the use of resins in in vitro systems of *Scutellaria* roots may be beneficial since it promotes extracellular storage of metabolites, which greatly facilitates their further isolation and purification at the end of the bioprocess.

The obtained results indicate, that in some cases the use of stress-inducing biotic factors may be one of the methods of inducing flavonoid biosynthesis in transformed root cultures of the species of the genus *Scutellaria*. In a previous study, stress conditions induced by yeast extract and bacterial lysates stimulated acteoside and flavone biosynthesis in hairy root culture of *S. lateriflora*^22^.

Chitosan, which is a deacetylated form of chitin, was used in the presented study. It shows not only elicitor properties, but also increases the permeability of cell membranes and stimulates the synthesis of jasmonic acid. In this work, jasmonic acid was also investigated due to its well-known stimulating effect on the level of secondary metabolites and biosynthesis of new compounds^50–54^. Chitosan elicitation increased the production of wogonoside in the hairy roots of *S. lateriflora*. On the other hand, the control samples neutralized with acetic acid contained wogonin as the dominant compound, but its concentration was similar to that of the parent culture (Fig. 6). Based on the experiments conducted, it can be assumed, that elicitation with chitosan stimulates the production of the glycosidic form of the above-mentioned metabolite. The results of the conducted experiments with chitosan are the first data obtained for *S. lateriflora* using this elicitor.

Previously, Gharari et al.^35^ revealed that treatment with chitosan (50, 100 and 200 mg/L) and chitosan in combination with methyl jasmonate (100 µM) had a significant influence on the accumulation of flavonoids in hairy root culture of *S. bornmuelleri,* while MeJa used alone was ineffective. The use of 100 mg/L chitosan gave the total content of chrysin of 52.34 µg/mg, wogonin of 19.5 µg/mg and baicalein of 42.3 µg/mg and these contents are approximately 8.5-, 7.6- and sevenfold compared with the control. Interestingly, chitosan showed synergistic effect with MeJa. The combination of 50 mg/L of chitosan with MeJa at 100 µM, resulted in the total content of these compounds of 56.47, 27.26 and 79.69 µg/mg, respectively which was about 9, 10.6 and 13.3 times higher compared the control. In the hairy roots of *S. bornmuelleri,* the combination of chitosan and methyl-*β*-cyclodextrin (*β*-CD) did not affect the level of flavone accumulation, in contrast to *β*-CD used alone^35^. Furthermore, when the hairy roots of *S. lateriflora* were elicited with methyl-*β*-CD (15 mM) for 24 h and grown in darkness, the wogonoside content of the elicitated cultures was higher, while the same treatment and cultivation with access to light resulted in an increase wogonin level, which confirms the thesis, that light is an important environmental factor affecting flavonoid biosynthesis^26^.

Similarly to chitosan, elicitation with jasmonic acid enhanced the production of wogonoside in *S. lateriflora* hairy roots (Fig. 7). It is worth noting that, in contrast to jasmonic acid, in our study (unpublished data) methyl jasmonate was found to be ineffective due to poor biomass growth and low content of the analyzed compounds, the phenomenon also previously revealed by Marsh et al.^18^. It can therefore be assumed that the transformation of MeJa to jasmonic acid did not take place in the studied root culture. However, the results of the experiments using MeJa in the in vitro cultures of *Scutellaria* sp. are inconclusive. In the study of Tuan et al*.* under the stress induced by exogenous methyl jasmonate, transcriptional regulation of genes responsible for flavonoid biosynthesis was observed, which resulted in an increase in the content of baicalin, baicalein, and wogonin in the *S. lateriflora* hairy roots. However, the accumulation of wogonin varied over time – it increased dramatically 6 h after elicitation, decreased until 48 h, and then increased again to 96 h. Wogonoside content was not evaluated^29^. The stimulating effect of MeJa on the level of baicalin, baicalein, and wogonin has also been demonstrated for hairy root cultures of *S. baicalensis*^37,55,56^. As mentioned above, MeJa was also an ineffective elicitor of hairy root culture of *S. bornmuelleri*.

Although the levels of wogonin and wogonoside in hairy roots grown in agitated shake flask cultures under standard conditions were comparable (11.5 mg/g DW and 12 mg/g DW, respectively)^22^, the production profile changed after the biomass was transferred to the bioreactor. In the bioreactor-grown roots, wogonoside was the dominant metabolite (34.9 mg/g DW). Moreover, the concentrations of flavones, which were not identified or were present in small amounts in *S. lateriflora* biomass grown in Erlenmayer flasks (scutellarin, baicalein, chrysin), increased after the culture was scaled-up. It should be noted, however, that among *Scutellaria* species, *S. lateriflora* is not the richest source of these compounds, unlike e.g. *S. baicalensis*^57,58^.

Finally, it was decided to conduct an experiment with *P. carotovorum*, serving as a biotic elicitor, for *S. lateriflora* grown in a basket-bubble bioreactor. As it was shown earlier (Wilczańska-Barska et al.^22^), in an analogous experiment conducted with the use of agitated cultures of hairy roots of the plant, the biosynthesis of flavonoids was shifted towards the production of wogonin, whose content reached approximately 3% DW. Since wogonin has been shown to exhibit a wide range of bioactivity, including antiviral, anti-inflammatory, and anticancer^7,12,59^, it was desirable to develop a system capable of selective biosynthesis of this metabolite. As in the case of shake flask culture, elicited on 26th day^22^, the bacterial elicitor was applied in the second half of the experiment. Due to the extension of the cultivation time of hairy roots in the bioreactor to 60 days (compared withthe agitated culture maintained for 40 days), the bacterial lysate was added on 40th day. Bioreactor-grown hairy roots treated with *P. carotovorum* were characterized by a high content of wogonin (37.9 mg/g DW), which was almost 10 times higher than the level of wogonoside. Thus, it has been shown, that with the use of *P. carotovorum* elicitation strategy, it is possible to achieve a targeted biosynthesis of wogonin in the hairy roots of *S*. *lateriflora*, both in a shake culture and in a bioreactor. The study also demonstrated, that wogonin may possibly be a phytoalexin, i.e. a compound produced by plants under environmental stress.

To our best knowledge, the current work is the first study employing a bioreactor for the cultivation of *S. lateriflora* hairy roots. Previous scale-up studies on *Scutellaria,* have been conducted only on in vitro shoot cultures of different skullcap species. For instance, polyester-supported Liquid Culture System was proposed for growing microshoots of *S. lateriflora*, *S. costaricana* and *S. baicalensis*. The roots of *S. lateriflora* separated from the microshoots, showed a significantly higher concentration of wogonin than the microshoots (1.26 µg/mg and 0.524 µg/mg, respectively)^60^. Microshoot cultures of *S. lateriflora* with total content of flavonoids 17.83–23.48 mg/g DW and with dominant baicalin (13.88–21.91 mg/g DW) were also maintained in the temporary immersion bioreactor. Wogonin and wogonoside concentration was 0.68–1.69 and 0.53–1.74 mg/g DW, respectively^28^. In both studies, the data on the production of the tested compounds are not given.

In the other work, in the shoots of *S. alpina* grown in the nutrient sprinkle bioreactor, the wogonoside concentration reached 4.05 mg/g DW (6.7 mg/L)^61^. It should be emphasized, that the above-mentioned amounts of wogonin and wogonoside in the shoot cultures were much lower than those obtained in the presented research on hairy roots of *S. lateriflora* grown in the bioreactor. Moreover, in vitro shoot cultures require specific bioreactor types, such as temporary immersion systems, which are difficult to scale-up^62^. On the other hand, hairy root cultures offer numerous advantages, such as fast growth, genetic stability, and high concentrations of secondary metabolites. Moreover, they can be grown using a variety of bioreactor types^63–65^.

## Conclusions

The presented work is the first report on the influence of biotechnological strategies, such as culture model used, precursor feeding, chitosan elicitation or permeabilization, on the biosynthesis of biologically active flavones and acteoside in *S. lateriflora* hairy roots. The study demonstrated, that the production profiles of secondary metabolites differed depending on the strategy employed. In the shake flask culture, the addition of sodium cinnamate (1.0 mg/L) in the stationary phase of the culture was the most effective strategy to obtain high production of flavones, especially wogonoside. On the other hand, permeabilization with DMSO (10 µg/ml for 24 h) or methanol (30% for 12 h) provided high content of wogonin. Hairy roots cultivated in a bioreactor turned out to be a high-yielding source of wogonoside and after the addition of the bacterial lysate of *P. carotovorum*, targeted biosynthesis of wogonin was achieved. It is worth noting, that the concentrations of wogonin obtained in the current work (up to over 3% DW, depending on the strategy applied), noticeably exceed the content reported in roots of naturally- or greenhouse-grown *S. lateriflora* (up to ca. 0.1% DW), and other medicinally-important skullcaps such as *S. baicalensis* (ca. 0.1–1.0% DW)^66–68^. The preliminary studies on the cultivation of *S. lateriflora* hairy roots in bioreactor systems are a good starting point for further research on increasing the scale of biomass cultivation of this species, in order to obtain therapeutically valuable secondary metabolites. It should be noted, however, that the conducted experiments concerning the scaling-up of *S. lateriflora* hairy root cultures are preliminary and require further studies on process optimization.

### Supplementary Information

Supplementary Information.

## Data Availability

The datasets generated and analyzed during the current study are available from the corresponding author on reasonable request.

## Abbreviations

DMSO: Dimethyl sulfoxide
DW (g/L): Dry weight
FW (g/L): Fresh weight
Gf (%): Growth factor
