# Supplementary material for: Biotechnological strategies for controlled accumulation of flavones in hairy root culture of Scutellaria lateriflora L
Source: Sci Rep. 2023 Nov 21;13:20422. doi: 10.1038/s41598-023-47757-7 (PMC10663461; doi:10.1038/s41598-023-47757-7)
Supplement: Supplementary file 1 — Supplementary Information. [file 41598_2023_47757_MOESM1_ESM.pdf]

Supplementary materials

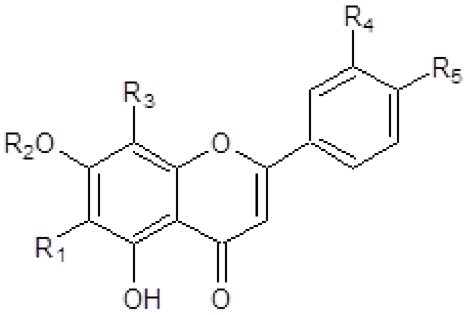

General structure of flavones

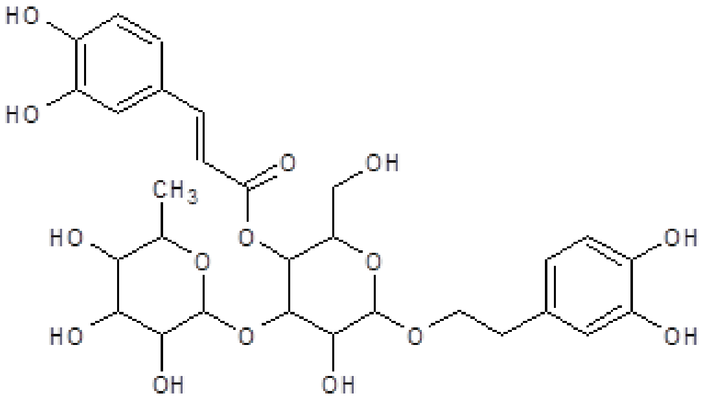

acteoside

Compound	R_1	R_2	R_3	R_4	R_5
**baicalin**	OH	*O*-glucuronic acid	H	H	H
**baicalein**	OH	H	H	H	H
**chrysin**	H	H	H	H	H
**scutelarein**	OH	H	H	H	OH
**scutelarin**	OH	*O*-glucuronic acid	H	H	OH
**wogonin**	H	H	OCH_3	H	H
**wogonoside**	H	*O*-glucuronic acid	OCH_3	H	H

Fig. S1. Chemical structures of acteoside and flavones present in *Scutellaria* genus.

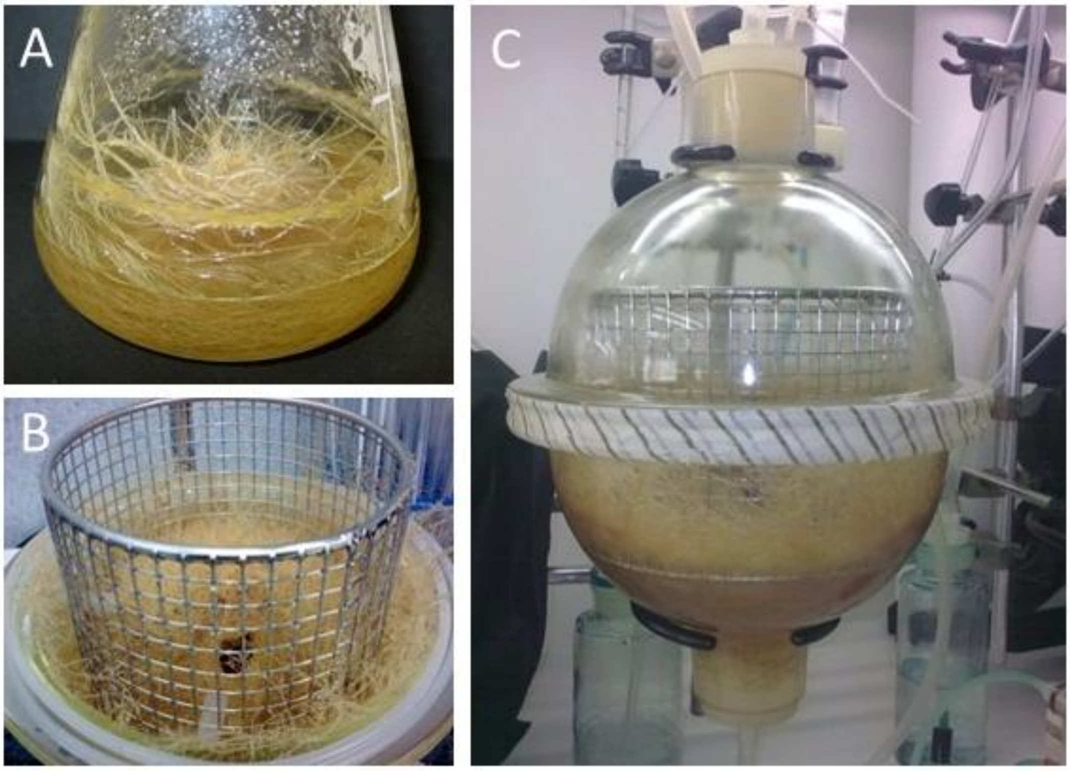

Fig. S2. Hairy roots of *Scutellaria lateriflora*: A) cultivated in darkness on ½ B5 medium in Erlenmeyer flasks (40th day of the growth cycle); B) and C) cultivated in darkness in a bubble-basket bioreactor (60th day of the growth cycle);

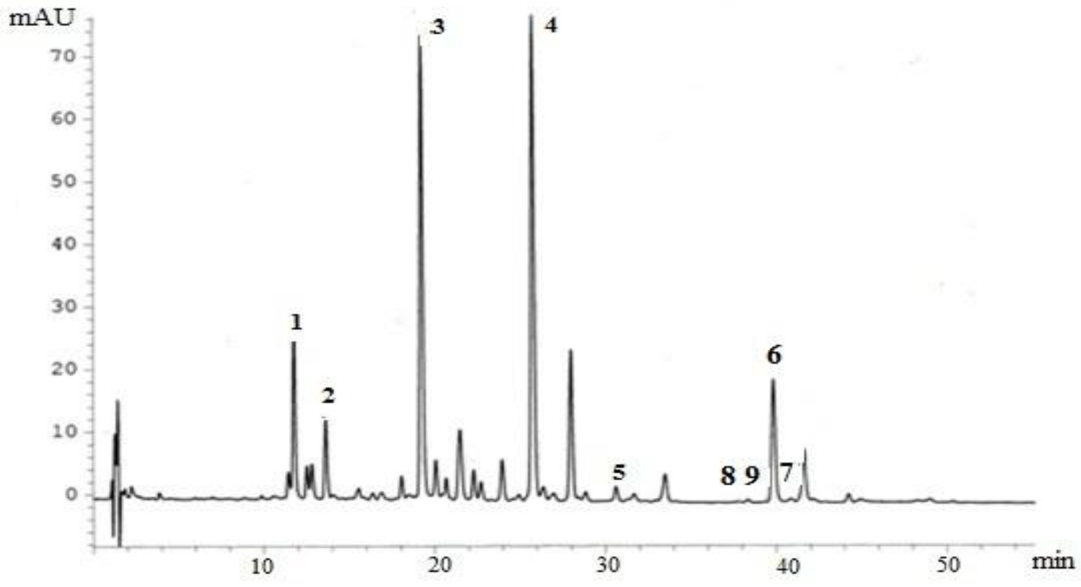

Fig. S3. HPLC chromatogram of a methanol extract from *S. lateriflora* hairy roots grown under standard conditions (40th day of the growth cycle). 1 – acteoside, 2 – scutellarin, 3 – baicalin, 4 – wogonoside, 5 – baicalein, 6 – wogonin, 7 – chrysin, 8 – 7-O-methylether of baicalein, 9 - 5,6,7-O-trimethylether of baicalein. (HPLC conditions: Wilczańska-Barska, A. et al., (2012) Biotechnology Letters, 34, 1757-1763.

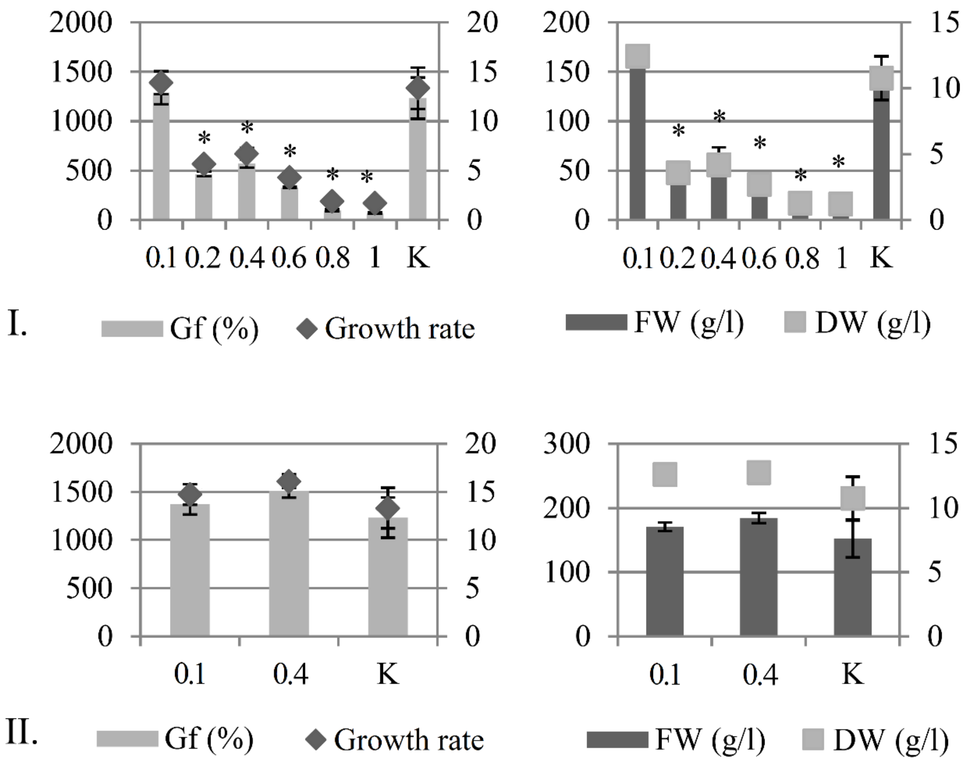

Fig. S4. The effect of phenylalanine added in the initial phase (I) (concentrations 0.1; 0.2; 0.4; 0.6; 0.8 and 1.0 mmol/l) and in the stationary phase (II) (concentrations 0,1 and 0.4 mmol/l) of *S. lateriflora* hairy root culture on growth of biomass, collected on the 40th day of culture (mean values, n=3 ± SD). The values marked with * are statistically significant compared to the control at $p < 0.05$. K – hairy roots grown under standard conditions (control).

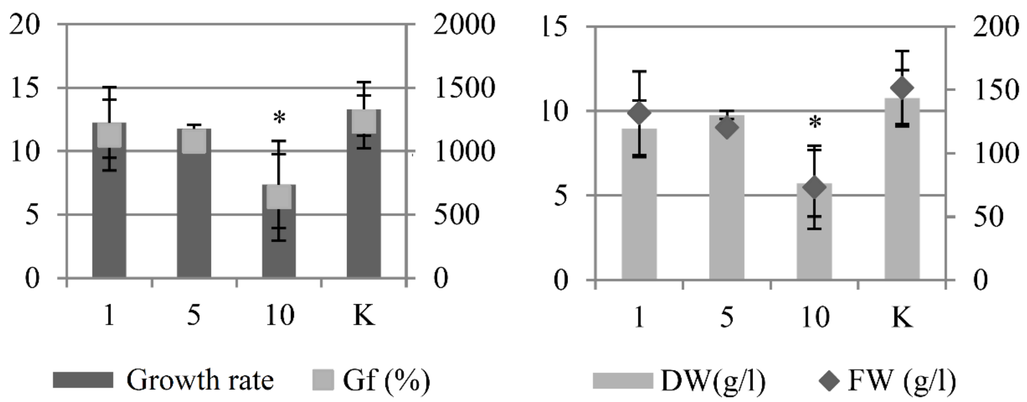

Fig. S5. The effect of cinnamic acid added in the initial phase (concentrations 1.0, 5.0 and 10.0 mg/l) of *S. lateriflora* hairy root culture on growth of biomass, collected on the 40th day of culture (mean values, n=3 ± SD). The values marked with * are statistically significant compared to the control at $p < 0.05$. K – hairy roots grown under standard conditions (control).

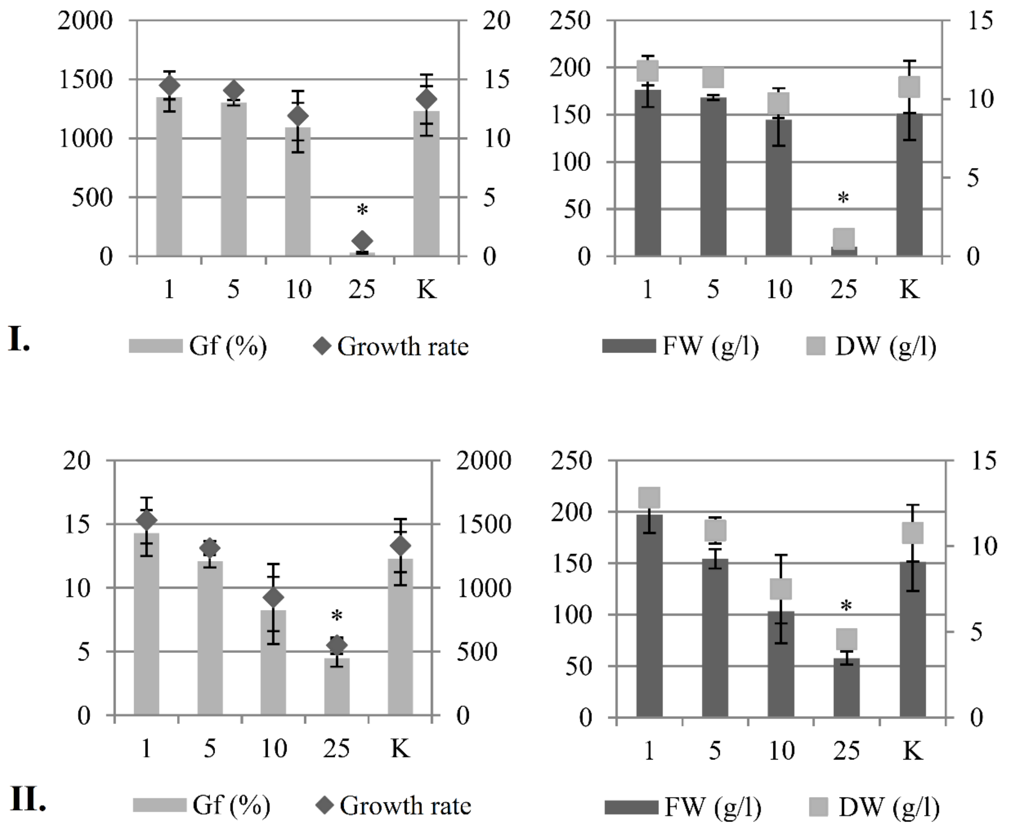

Fig. S6. The effect of sodium cinnamate added at concentrations of 1.0, 5.0, 10.0 and 25.0 mg/l in the initial phase (I) and in the stationary phase (II) of *S. lateriflora* hairy root culture on growth of biomass, collected on the 40th day of culture (mean values, n=3 ± SD). The values marked with * are statistically significant compared to the control at $p < 0.05$. K – hairy roots grown under standard conditions (control).

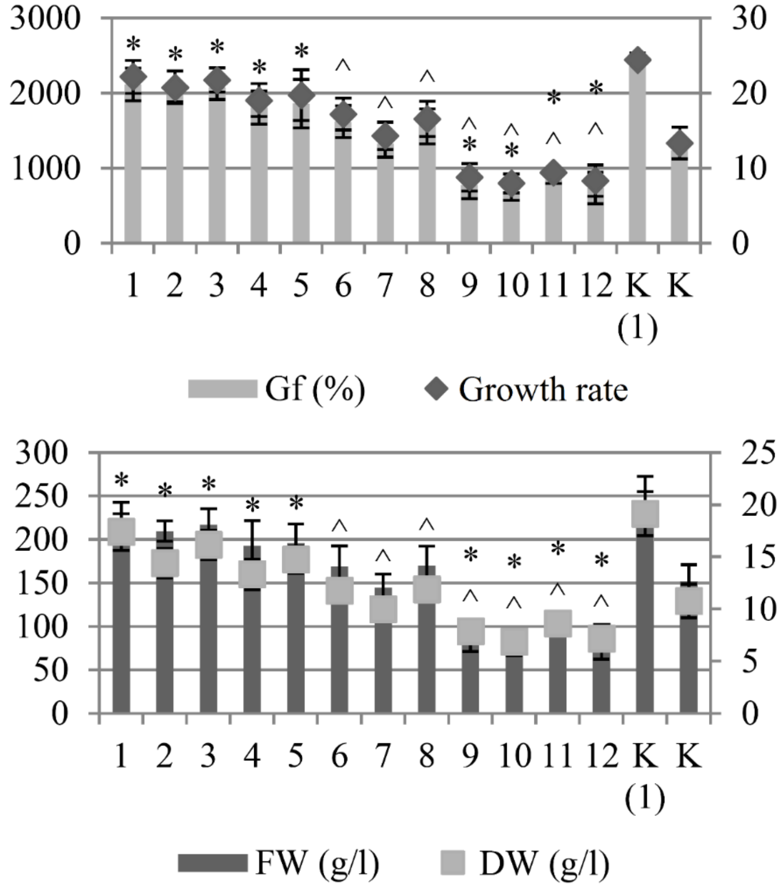

Fig. S7. The effect of permeabilizing factors, DMSO and methanol on growth of *S. lateriflora* hairy root culture (mean values, n=3 ± SD). The values marked with * are statistically significant compared to the control at p <0.05. The values denoted by ^ are statistically significant compared to the control with the exchanged medium at p <0.05. 1) and 2) DMSO at a concentration of 1 μg/ml for 12 and 24 hours, respectively; 3) and 4) DMSO at a concentration of 2 μg/ml for 12 and 24 hours, respectively; 5) and 6) DMSO at a concentration of 10 μg/ml for 12 and 24 hours, respectively; 7) and 8) methanol at a concentration of 10% for 12 and 24 hours, respectively; 9) and 10) methanol at a concentration of 20% for 12 and 24 hours, respectively; 11) and 12) methanol at a concentration of 30% for 12 and 24 hours, respectively; K (1) – hairy roots grown in a system with replacement of the culture medium; K – hairy roots grown under standard conditions (control).

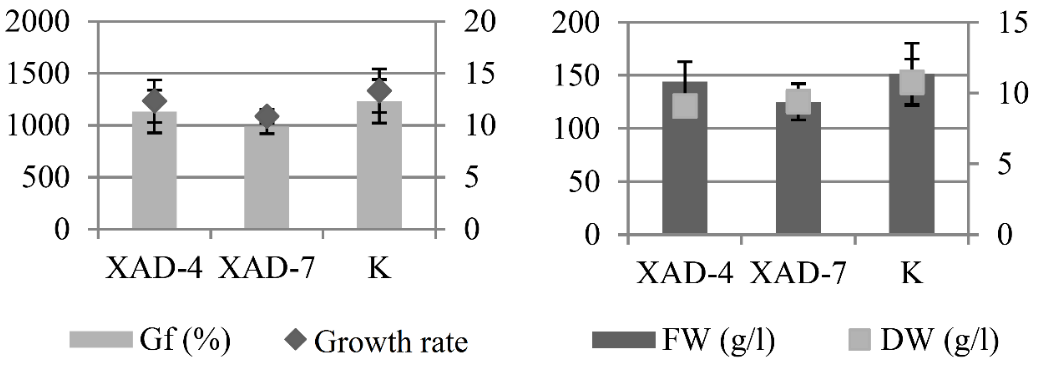

Fig. S8. The effect of Amberlite XAD-4 and XAD-7 resins used in the form of "mini-bags" at a concentration of 50 mg/50 ml on growth of *S. lateriflora* hairy root culture (mean values, n=3 ± SD). The values marked with * are statistically significant compared to the control at $p < 0.05$. K – hairy roots grown under standard conditions (control).

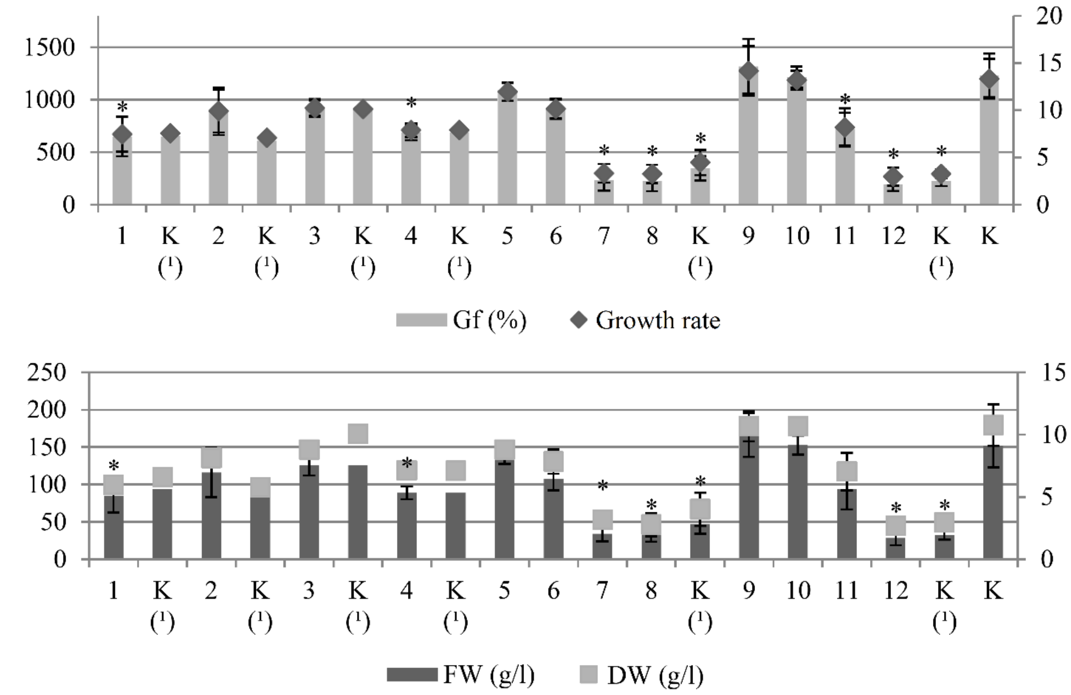

Fig. S9. The effect of chitosan added in the stationary phase of *S. lateriflora* hairy root culture on growth of biomass (mean values, n = 3 ± SD). The values marked with * are statistically significant compared to the control cultivated under standard conditions at $p < 0.05$. Chitosan added: at a concentration of 30 mg/l – biomass collected after 24 hours (1), 48 hours. (2), 72 hours (3), 96 hours (4), 7 days (5) and 14 days (9); at a concentration of 100 mg/l – biomass collected after 7 days (6) and 14 days (10); at a concentration of 200 mg/l – biomass collected after 7 days (7) and 14 days (11); at a concentration of 250 mg/l – biomass collected after 7 days (8) and 14 days (12); K (1) – hairy roots grown after addition of a specified volume of acetic acid neutralized with 1 N NaOH at the appropriate time of the experiment; K – hairy roots grown under standard conditions (control).

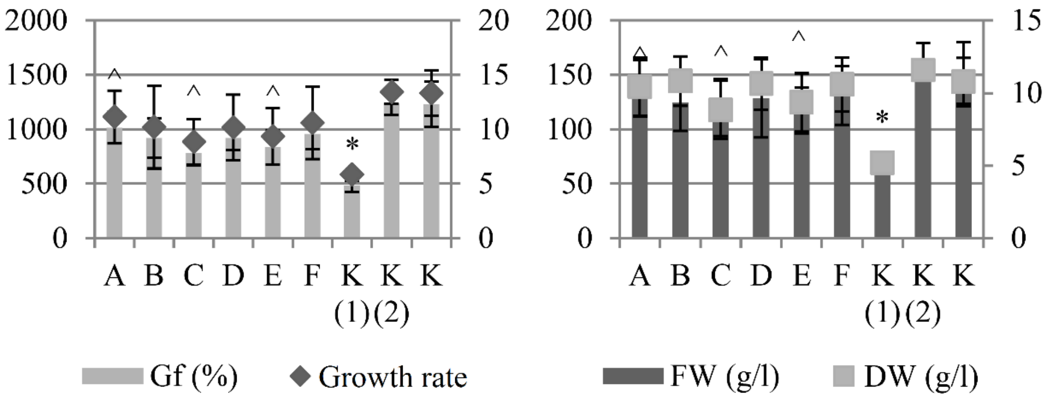

Fig. S10. The effect of jasmonic acid added in the stationary phase of *S. lateriflora* hairy root culture on growth of biomass (mean values, n = 3 ± SD). The values marked with * are statistically significant compared to the control cultivated under standard conditions at $p < 0.05$. The values marked with ^ are statistically significant compared to the control cultivated in the presence of ethanol at $p < 0.05$. Jasmonic acid added: at a concentration of 100 µmol/l – biomass collected after 7 days (A) and 14 days (B); at a concentration of 200 µmol/l – biomass after 7 days (C) and 14 days (D); at a concentration of 300 µmol/l – biomass collected after 7 days (E) and 14 days (F); K (1) – hairy roots grown in the presence of ethanol and collected after 7 days; K (2) – hairy roots grown in the presence of ethanol and collected after 14 days; K – hairy roots grown under standard conditions (control).

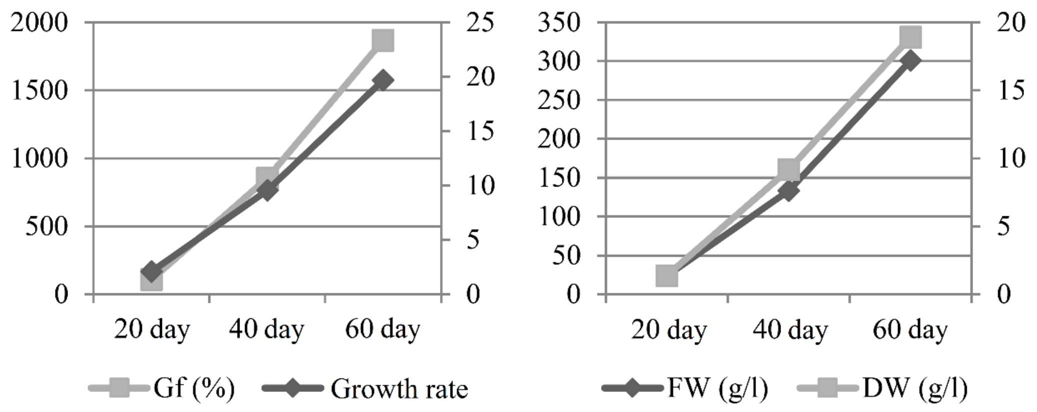

Fig. S11. Growth profile of *S. lateriflora* hairy roots cultivated in the dark in a basket-bubble bioreactor on ½ B5 medium liquid medium for 20, 40 and 60 days (n=1).

Tab. S1. The validation data for the determination of analyzed secondary metabolites by elaborated HPLC method.

Compound	Linearity		LOQ (µg/ml)	Repeatability (RSD, %)	Inter-day precision (RSD, %)	Intra-day precision (RSD, %)
	linear regression equation	R				
acteoside	f(x)=0.000489261x + 0.11242	0.9999	7.09	1.85	1.64	2.02
scutellarin	f(x)=6.24712e-5x + 0.745568	0.9999	2.80	0.10	0.20	0.12
baicalin	f(x)=6.32623e-5x + 0.0972131	0.9999	1.13	0.52	1.10	1.16
wogonoside	f(x)=5.79135e-5x – 0.154545	0.9999	1.06	0.30	0.32	0.32
baicalein	f(x)=4.67388e-5x + 0.178836	0.9999	0.71	0.10	0.34	0.42
wogonin	f(x)=0.000112308x – 0.159985	0.9999	0.99	0.20	0.11	0.10
chrysin	f(x)=4.17371e-5x – 0.0107353	0.9999	0.16	0.10	0.05	0.03

R – regression coeficient
LOQ – limit of quantification
RSD – relative standard deviation (%)

Tab. S2. The recovery of analyzed secondary metabolites by elaborated HPLC method (n = 3).

Comound	Amount present in analyzed sample (mg)	Amount added to the sample (mg)	Amount found (mg) ± SD	Recovery ± SD (%)	Mean recovery (%)
acteoside	3.190	1.595	5.264 ± 0.067	110.00 ± 1.41	110.00
	3.190	2.393	6.169 ± 0.198	110.50 ± 3.54	
	3.190	3.988	7.860 ± 0.254	109.50 ± 3.55	
scutellarin	0.298	0.149	0.426 ± 0.023	98.60 ± 0.71	101.85
	0.298	0.224	0.525 ± 0.070	100.60 ± 1.27	
	0.298	0.373	0.714 ± 0.018	106.35 ± 2.62	
baicalin	2.624	1.312	3.912 ± 0.105	99.37 ± 2.67	100.30
	2.624	1.968	4.689 ± 0.057	102.11 ± 1.23	
	2.624	3.280	5.870 ± 0.298	99.43 ± 5.06	
wogonoside	1.572	0.786	2.319 ± 0.103	98.35 ± 4.38	101.04
	1.572	1.179	2.738 ± 0.182	99.53 ± 6.63	
	1.572	1.965	3.723 ± 0.038	105.25 ± 1.06	
baicalein	0.228	0.114	0.340 ± 0.004	99.41 ± 1.41	99.77
	0.228	0.171	0.401 ± 0.009	100.37 ± 2.29	
	0.228	0.285	0.511 ± 0.035	99.52 ± 6.76	
wogonin	0.483	0.242	0.803 ± 0.010	110.76 ± 1.36	109.40
	0.483	0.362	0.919 ± 0.014	108.76 ± 1.68	
	0.483	0.604	1.182 ± 0.006	108.69 ± 0.59	
chrysin	0.003	0.0015	0.00495 ± 0.000	110.00 ± 1.41	110.25
	0.003	0.0023	0.00585 ± 0.000	110.35 ± 2.62	
	0.003	0.0038	0.00751 ± 0.000	110.44 ± 3.54	

Tab. S3. Content (% DW) Production (mg/L) and productivity (mg/L/day) of secondary metabolites in *S. lateriflora* hairy roots grown in shake flasks and basket-bubble bioreactor, cultivated under standard conditions, and after elicitation with *Pectobacterium carotovorum*.

Compound	Experimental strategy											
	Shake flask culture[a]			Elicited shake flask culture[b]			Bioreactor culture[c]			Elicited bioreactor culture[d]		
	% DW	mg/L	mg/L/day	% DW	mg/L	mg/L/day	% DW	mg/L	mg/L/day	% DW	mg/L	mg/L/day
Acteoside	1.85	227.2	5.09	-	-	-	1.56	294.26	4.90	1.08	217.61	5.29
Scutellarin	0.06	7.1	0.18	0.02	2.0	0.05	-	-	-	0.24	48.59	0.81
Baicalin	1.45	180.3	4.33	-	-	-	0.82	154.7	2.58	0.66	133.60	2.23
Wogonoside	1.2	147.1	3.88	-	-	-	3.49	659.44	10.99	0.40	80.97	1.35
Baicalein	-	-	-	-	-	-	-	-	-	0.21	42.51	0.71
Wogonin	1.15	142.0	3.55	2.94	386.75	9.67	0.62	117.44	1.96	3.79	761.17	12.67
Chrysin	0.01	1.3	0.04	0.03	4.56	0.114	0.015	2.84	0.05	0.04	8.10	0.14
Sum of flavones[e]	3.86	477.8	12.0	2.55	393.31	9.83	4.94	934.42	15.57	5.34	1292.55	17.91

[a] biomass grown for 40 days; data according to (Wilczańska-Barska et al. 2012)
[b] biomas grown for 40 days, elicited with 15 ml/l of autoclaved *P. carotovorum* suspension (OD_{550} 1,75 = 21 x 10^8/ml) on 26th day; data according to (Wilczańska-Barska et al. 2012)
[c] culture grown in basket-bubble bioreactor for 60 days
[d] culture grown in basket-bubble bioreactor for 60 days elicited with 15 ml/l of autoclaved *P. carotovorum* suspension (OD_{550} 1,75 = 21 x 10^8/ml) on 40th day
[**e**] the sum of six flavones: baicalin, wogonoside, wogonin, baicalein, scutellarin and chrysin
